# Supplementary material for: Feasibility of Leukemia-Derived Exosome Enrichment and Co-isolated dsDNA Sequencing in Acute Myeloid Leukemia Patients: A Proof of Concept for New Leukemia Biomarkers Detection
Source: Cancers (Basel). 2022 Sep 16;14(18):4504. doi: 10.3390/cancers14184504 (PMC9497185; doi:10.3390/cancers14184504)
Supplement: Supplementary file 1 [file cancers-14-04504-s001.zip › cancers-1859546-supplementary.pdf]

In order to select the best isolation kit for our purpose, 4 samples were treated in parallel with a commercial kit for total plasmatic exosomes isolation (Invitrogen) and with a commercial kit providing an enrichment in exosomes derived from malignant cells (Exosomics Siena S.p.A).

Exosomal dsDNA Quality Control (QC) analysis was based on the evaluation of 230/260 and 260/280 ratio by Nanodrop (Thermofisher Scientific) and on a fluorimetric method using Quant-iT PicoGreen dsDNA Assay kit (Thermo Fisher Scientifics).

The results of the QC are reported in the table below.

Sample	***SeleCTEV (Enrichment)***		***Total Exosome Isolation (Total)***	
**1**	*Quantification*	*9.3 ng/ul*	*Quantification*	*6.2 ng/ul*
	230/260	*1.91*	*230/260*	*0.90*
	260/280	*1.83*	*260/280*	*1.11*
**2**	*Quantification*	*6.4 ng/ul*	*Quantification*	*4.9 ng/ul*
	230/260	*1.96*	*230/260*	*0.99*
	260/280	*1.78*	*260/280*	*2.16*
**3**	*Quantification*	*4.6 ng/ul*	*Quantification*	*2.1 ng/ul*
	230/260	*1.98*	*230/260*	*0.75*
	260/280	*1.85*	*260/280*	*0.98*
**4**	*Quantification*	*9.7 ng/ul*	*Quantification*	*4.4 ng/ul*
	230/260	*2.01*	*230/260*	*0.95*
	260/280	*1.79*	*260/280*	*2.21*
